# The Role of Built Environment on Health of Older Adults in Korea: Obesity and Gender Differences

**DOI:** 10.3390/ijerph16183486

**Published:** 2019-09-19

**Authors:** Mi Namgung, B. Elizabeth Mercado Gonzalez, Seungwoo Park

**Affiliations:** Department of Urban Engineering, Pusan National University, Busan 46241, Korea; elyzmglez@gmail.com (B.E.M.G.); tmddn26118@naver.com (S.P.)

**Keywords:** obesity, built environment, gender difference, spatial analysis, Korea

## Abstract

This study examines the effect of the built environment on obesity in older adults, taking into consideration gender difference. In this regard, we ask two questions: (1) How does the built environment affect obesity in older adults? (2) Is there a gender difference in the effect of the built environment? To examine the research questions, this study uses the 2015 Korean National Health and Nutrition Survey and geographically weighted regression (GWR) analysis. The empirical analyses show that environmental factors have stronger effects on local obesity rates for older men than for older women, which indicates a gender difference in obesity. Based on these findings, we suggest that public health policies for obesity should consider the built environment as well as gender difference.

## 1. Introduction

The increasing rate of obesity in Korea, as well as in western countries, is a growing public health concern. Obesity is currently a serious health problem and a major risk factor for various health issues, including heart disease, as well as other socially and financially negative consequences [1,2]. In 2016, the World Health Organization (WHO) declared obesity as one of the top ten risk factors in the world, with more than 1.9 billion adults at risk, and with over 650 million adults classified as being obese [3]. Obesity is caused by the imbalance of caloric/energy consumption and caloric/energy expenditure, which indicates an imbalance in consumption as related to physical inactivity [1,2]. Moderate physical activity during leisure time can help improve health and weight control [2]. Obesity is also associated with increased likelihood of psychological disorders, such as depression [4]. Among the various studies that have focused on obesity, there is widespread belief that sedentary behaviors are one of the primary causes that may result in obesity in children and adults alike [5,6,7,8,9]. Sedentary behavior is affected by the individual’s surrounding built environment and the individual’s socio-demographic characteristics [5,6,7,8,9].

In Korea, according to the 2016 Korea Centers for Disease Control and Prevention (KCDC), the prevalence of obesity, defined as a body mass index (BMI) ≥ 25 kg/m^2^, is about 40.2% (39.7% for men and 40.7% for women) in adults over the age of 65 [10]. This means that more than one-third of older Korean adults are obese. Korea has become an “aged society” since 2018, meaning that the population aged 65 or older exceeds 14% of the total population [11]. In terms of older adults, generally after the age of 60 years old, weight and BMI remain unchanged [12]. Furthermore, older adults who suffer from depression are less likely to engage in physical activity, which in turn increases the risk of obesity [13]. With the number of people aged over 65 making up 14% of the population, it is imperative to research the causes for rapid increase in obesity rates, in order to prevent other negative health consequences.

There is a growing consensus among researchers that, rather than biology/genetics, the environment is what generates obesity [8,14]. Researchers agree that biology/genetics influence an individual’s weight and height. However, the rapid increase in obesity development, and the prevalence in recent decades, can be attributed to personal, behavioral, and environmental factors, rather than biology/genetics as the principal causes [8,14]. *The Thesis of Obesogenic Environment* proposes that the built environment can both facilitate or hinder an individual’s healthy behavior [15,16]. Therefore, obesity prevalence rates can partly be attributed to the level of exposure to healthy environments that promote healthier dietary choices and encourage physical activity [2,16,17,18]. These statements indicate that individual behaviors, such as eating habits and physical activity/inactivity are influenced by environmental factors [2,15,19,20]. A number of studies have been conducted on the association between the built environment and obesity in developed countries, such as the United States and other European countries [21,22,23,24,25,26,27,28,29,30]. These studies indicate that the built environment has a significant impact on obesity. More specifically, neighborhoods with increased numbers of green areas, recreational areas and high street connectivity are associated with lower prevalence of obesity [28,29,31]. A number of studies have focused on the influences of the built environment on physical activity [25,32,33,34,35,36,37,38,39,40]. These studies suggest that the characteristics of the built environment, such as the availability of parks and green space, and land-use mix promote physical activity, such as walking and exercising [25,32,33,34,35,36,37,38,39,40]. People who are more likely to meet the physical activity recommendations are more likely to engage in physical activity, and thus decrease their risk of being overweight/obese [34,41]. Furthermore, it is important to consider the level of neighborhood socioeconomic status (SES) to assess the influence of the built environment on obesity [15]. People of higher economic status have more resources in terms of choosing healthier foods and participating in further physical activity and people with higher levels of education are also more likely to integrate healthier behaviors and habits into their lifestyle [17,42,43].

In previous studies, research on neighborhood characteristics and its influence on older adults has not been sufficiently explored. Given that older adults spend more of their time in their neighborhoods, they are more likely to use their neighborhood environment, as compared to younger adults [44]. Thus, the study of the built environment in relation to obesity for older adults is important, as older adults are far more inclined to be affected by their neighborhood environment. There have been more studies recently which have researched the effects of neighborhood walkability on obesity in older adults. Results from the studies suggested that higher walkability and/or income is associated with a greater likelihood of meeting the recommendation levels of physical activity [45,46]. Li et al. [45] study showed that in older adults, there was a significant interaction between neighborhood walkability and physical activity. These results show that residing in highly walkable neighborhoods is associated with a decrease in BMI. However, these results were applicable to only older adults engaging in high intensity physical activity, not to those engaging in moderate physical activity [45]. King et al. [46] also showed that older adults residing in highly walkable neighborhoods and engaging in moderate to physical activity had lower BMI. Lawton’s model states that if an individual’s capabilities are not on par with the environmental demands, this can cause inactivity in older adults. In agreement with this model, a recent study on older adults showed that the majority of overweight and obese older adults were worried about neighborhood safety in terms of physical injury, such as falling down when walking [47]. Another study showed the strongest determinants for walking in overweight and obese older adults were related to education level. People with an education level lower than university were more than five times less likely to be active [47].

The effect of the built environment on obesity in older adults may differ by gender. There was a significant difference in the level of physical activity and walking between older men and women [48]. Studies found that older men were more likely to engage in physical activity than older women, whereas older women were more likely to engage in household activities than older men [49,50]. This suggests that older men spend more time outdoors in neighborhoods and are less likely to be obese through increased physical activity. In addition, older women are more likely to perceive barriers to physical activity than older men [48]. Given that perceived barriers to exercise are negatively associated with physical activity among older adults [51], older men are more likely to participate in outdoor physical activities. These findings suggest that older men’s obesity may be more associated with the built environment than older women’s obesity. Another difference between older men and women is the length of trip. Older women tend to take shorter trips compared to older men [52]. This suggests that older men spend more time around their neighborhoods and engage in more outdoor activities, and thus, older men are more likely to be affected by the built environment. Furthermore, studies also suggest a stronger relationship between built environment and obesity for older men than for older women [31]. For instance, Araújo et al. [53] found that better street connectivity and intermediate percentage of local commerce were associated with a lower risk of obesity for older men, but not for older women. 

Recently, with increasing interest in public health issues in Korea, most of the studies have focused on the association between the environment and health [54,55,56] and between the environment and physical activity [57,58,59]. However, few studies have examined the effect of the environment on obesity, particularly for older adults. Furthermore, studies based on obesity mostly focus on case studies conducted in Seoul, but not on a national basis [54,56,57,58,59]. Therefore, this study attempts to examine the role of built environment on obesity for older adults, by also considering gender differences in Korea. In this regard, we ask the following research question: Is there a gender difference in the effect of built environment on obesity in older adults? Based on the theoretical connections between the built environment and local obesity with gender difference in older adults, we propose two hypotheses:

H1: There is a greater effect of environmental factors on local obesity for older men than for older women.

H2: There is more varying effect of environmental factors on local obesity for older men than for older women.

## 2. Materials and Methods 

### 2.1. Materials

This study aims to examine the effect of the built environment on obesity for older adults and gender difference. For empirical analysis, we utilized the data provided by the “2015 Korea Community Health Survey” (KCHS) which is a community-based nationwide survey initiated in 2008 by Korea Centers for Disease Control and Prevention. The sample size of the KCHS data is 228,558, which includes 102,829 men and 125,729 women. In this study, the sample size is 13,201, which includes 7619 female respondents and 5582 male respondents, all aged ≥ 65 years old. The questionnaire covers a variety of topics to assess the personal health of the respondents (e.g., weight, height, physical activity, smoking, alcohol use, weight control, quality of life, and medical service). The dependent variable in this study is local obesity rate. To measure the local obesity rate for older men and women, we first defined the body mass index (BMI), a simple index of weight (kilograms) for height (meters squared), which is commonly used to classify underweight, overweight and obesity in adults. As defined by the World Health Organization (WHO), overweight adult is someone with a BMI of 25 to 29.9 and obese adult is someone with a BMI of 30 or higher [3]. However, as Asian countries are generally considered to have lower BMI scores [3,19,31,41,60], we considered people with BMI scores of 25 and higher as obese. After calculating the BMI scores of the respondents in separate groups of older men and women, we calculated the local obesity rates—number of obese people (BMI ≥ 25 kg/m^2^)—to the number of respondents in each locality for older men and women. The administrative divisions of Korea broadly consist of upper-level and lower-level municipalities. Since the lower-level municipalities are more intimately related to the local environment that influences obesity, we use data collected for the local environment at the lower-level municipality. While there are 228 lower-level municipalities in Korea, we excluded 11 localities located on islands where the data were not available. As a result, from the total of 228 localities, we used 217 localities for this study’s empirical analysis. 

To examine how environmental factors are differently associated with local obesity rates between older men and women, we included built environmental factors by reviewing previous studies. We included the number of fast-food restaurants per 1000 people, the number of sports facilities per 1000 people, the level of land-use mix, intersection density, and employment density as physical environment factors. The number of fast-food restaurants was per 1000 people [1,2,16,31,45,61,62,63,64].

The level of land-use mix was computed by the method of Bhat and Guo [65]:

Li=1−{ |rL− 13|+|mL−13|+|oL−13|43 },

where L_i_ is the mixed land-use index, L is the total land size, r is the size in residential land-use, m is the size in commercial/industrial land-use, and o is the size in other land-uses. According to the equation, this variable ranges from 0 to 1, where 0 indicates perfect homogeneity and 1 indicates perfect diversity in land-use.

The number of sports facilities per 1000 people [1,19,31,47,60,66] may promote physical activity and directly influence obesity rate. The level of land-use mix [1,15,16,21,24,34], intersection density [2,12,15,16,25,34,45], and employment density [2,19,34,41,43,47] can potentially improve walkability, which may reduce obesity rates. In addition, we included socio-economic factors, such as percentage of high school graduates and percentage of basic living recipients. Percentage of high school graduates was included because people with higher education levels are more likely to consider a healthy lifestyle [2,7,12,19,24,36,41]. Percentage of basic living recipients was included to reflect the level of poverty that increases obesity rates [2,6,15,16,20,25,36].

We collected most of our data for the study from the Korean Statistical Information Service (KOSIS) and used the data from 2015. The data regarding sport facilities, land-use, employment density, education level, and basic living recipients, were collected from the Korean Statistical Information Service (KOSIS). The data regarding fast-food restaurants and intersection density were obtained from Small Business Corporation and Korea National Spatial Data Infrastructure, respectively.

### 2.2. Methods

Our hypothesis suggests that environmental factors have a stronger and more varying effect on obesity rates for older men than for older women. To test these hypotheses, the analysis was conducted using a geographically weighted regression (GWR) model. This statistical method was used to test the associations between the dependent variable and the independent variables. GWR tests these associations and generates a basic regression model for each location to measure the geographic variability of the dependent variable (BMI) relative to the independent variables.

The geographically weighted regression (GWR) model’s unique spatial statistical analysis technique compares non-stationary variables (e.g., socio-economic factors, built environment characteristics), and shows the relationships between these from location to location [67]. GWR explains how our dependent variable, BMI, differs from every location in the country and changes between older men and women.

A GWR follows the equation:

$$y_{i}=\beta_{0}\left(u_{i},v_{i}\right)+\underset{k}{\sum }\beta_{k}\left(v_{i},\;v_{i}\right)x_{ik}+e_{i}$$

where $y_{i}$ is the dependent variable for observation i , $x_{ik}$ is the value of an independent variable k for obeservation i , $\left(u_{i},v_{i}\right)$ is the location of observation i , and $e_{i}$ is the error point at i . In GWR, a weighting scheme based on each individual location’s spatial proximity to a location i is applied to a specific location (localities in our case) to assign weights. In other words, near locations have more impact on the calibration of coefficients than far locations do, and thus GWR captures local influences of environmental factors on obesity when spatial heterogeneity is present. 

## 3. Results

Table 1 shows summary statistics for all variables used in this study. Table 1 suggests that across all regions in Korea, there is substantial variability in their values. Table 2 shows results from global regression (ordinary least squares, OLS) and GWR. The global regression analysis is utilized to examine the simultaneous effects of the built environment on local obesity. As shown in Table 3, there are differential effects of environment factors on local obesity between older men and women. More specifically, the number of fast-food restaurants per 1000 people, the number of sports facilities per 1000 people, the percentage of high school graduates, and the percentage of basic living recipients, are statistically significant on local obesity rates for older men, whereas the percentage of high school graduates and the percentage of basic living recipients, are significant for older women. It is important to consider that the global regression model assumes that the association between the built environment and local obesity are identical in a region and thus cannot be used to explore local variations in regression model coefficients. A GWR estimates locally varying coefficients of independent variables, as well as calculating mean values of the locally varying coefficients. The growth in the R^2^ in the GWR model explains a better fit to the data, compared to global regression. More specifically, for older men, the R^2^ is 0.35 in GWR, while it is 0.31 in the global regression. For older women, the adjusted R^2^ is 0.26 in GWR compared to 0.17 in the global regression. In addition, the Akaike information criterion (AIC) is a powerful tool for comparing different models that have the same independent variable [68,69,70]. 

**Figure 1:**
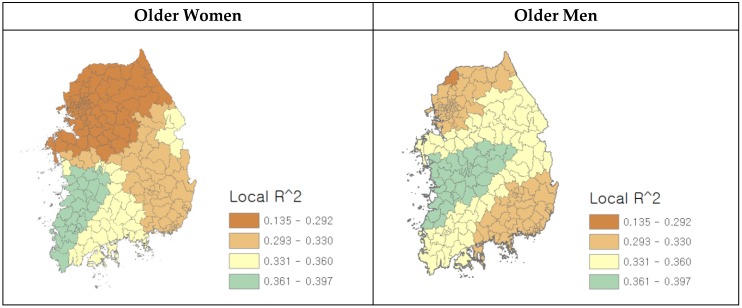
GWR model performance for older women and men: distribution of local R^2^ values.

The model with the lowest value AIC is considered the best model when compared to two or more models [68,69,70]. The AIC score for GWR is smaller than the AIC score from the global model for both older men and women, which indicates that the local model provides a better fit to the data. The spatial distribution of local R^2^ generated by GWR is presented in Figure 1. The maps show that the degree to which the environmental factors explain local obesity varies from one place to another in Korea and differs for older men and women. Local R^2^ in the west region is higher than the other regions, for both older men and women. In the east region, local R^2^ for older men is higher than for older women. Overall, there are more localities with higher local R^2^ values for older men, compared to older women. Absolute mean values of local coefficients indicate the overall impact of environmental factors on local obesity rates in Table 2. The higher mean value of local obesity rates for older men suggests that local obesity rates, overall, are greater for older men than for older women. The absolute mean values of four factors (number of fast-food restaurant per 1000 people, number of sports facilities per 1000 people, level of land-use mix, and percentage of basic living recipients) were higher for older men than for older women. Even though the absolute mean values of two environmental factors (intersection density and percentage of college graduates) were greater for older women, the absolute mean values of the other four environmental factors were higher for older men. Overall, these results support our first hypothesis that there is a greater effect of environmental factors on local obesity for older men than for older women. In Table 3, additional GWR results, including means and standard deviations of local coefficients, and minimum and maximum coefficients and their ranges for all older men and women can be found.

**Figure 2:**
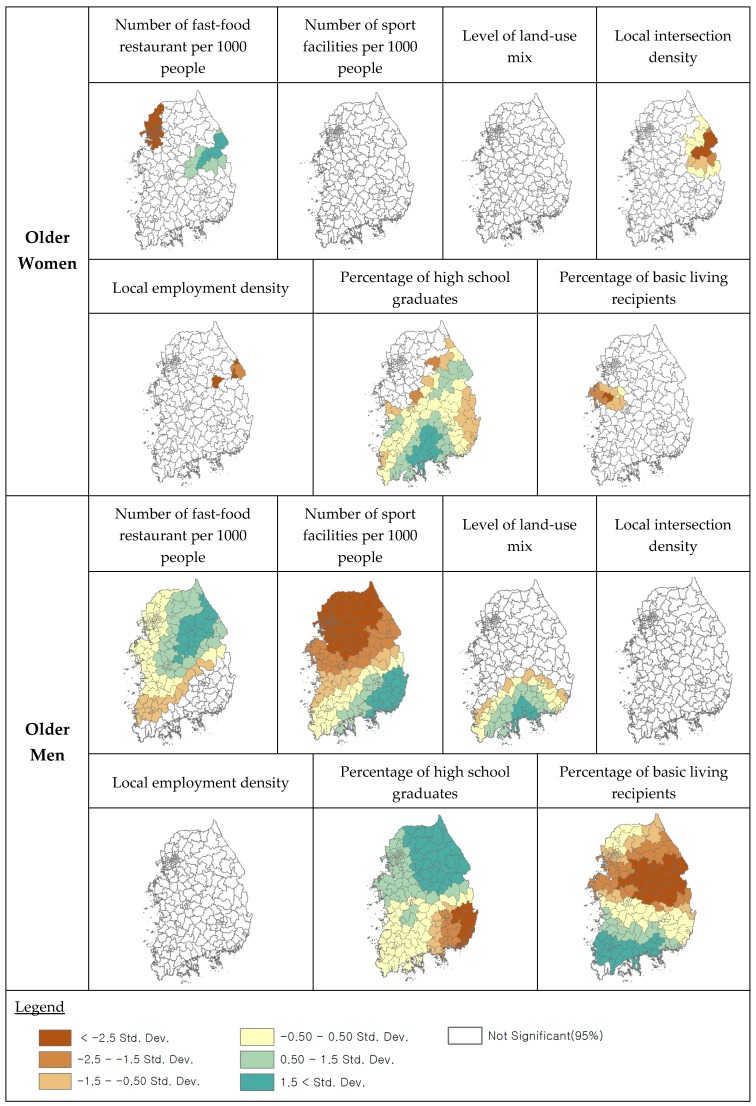
Localities with statistically significant local coefficients in GWR.

Table 4 compares the number of localities with significant coefficients for each of the environmental factors at a level of 95% between the older men and women. There were more localities (number of fast-food restaurants per 1000 people, number of sports facilities per 1000, level of land-use mix, percentage of high school graduates, and percentage of basic living recipients) for older men than for older women, that were significant environmental factors on local obesity rate. For the older women, there were more localities in employment density and intersection density that were significant environmental factors, as compared to older men. Figure 2 also shows the levels of regional variation for each of the environment variables for both older men and women. The environmental factors had higher levels of regional variation for older men than for older women. For example, local coefficients of the number of sports facilities per 1000 people were statistically significant in all localities for older men with standard deviations ranging from − 2.5 to over 1.5, whereas there is no statistical significance in any locality for older women. More specifically, the number of sport facilities per 1000 people demonstrated a strong positive relationship in the southeast region, a strong negative relationship in the north region, and a weak relationship in the central region, for older men. While local coefficients of the number of fast-food restaurant per 1000 people were statistically significant in most regions with standard deviations ranging from − 2.5 to 1.5 for older men, they were statistically significant in the east and northwest regions with standard deviations ranging from − 2.5 to − 1.5 and over 0.5 for older women. Overall, the associations between environmental factors and local obesity rates are spatially varying more for older men than for older women. As the standard deviations of the local significant coefficients are larger for older men than for older women, these results support our second hypothesis, that there is a more varying effect of environmental factors on local obesity for older men than for older women.

## 4. Discussion

Obesity has long been considered as one of the primary causes of various health problems. Numerous studies have examined factors affecting obesity and found that the built environment plays an important role in affecting obesity. However, not many studies have examined the influence of the built environment on obesity for older adults. Our study fills in the gap in the literature by examining not only the effect of the built environment on obesity for older adults, but also its gender differences.

By analyzing data from the 2015 Korea Community Health Survey, this study examined the associations between built environment and obesity for older men and women. Our study supports previous studies’ findings that demonstrate the importance of the built environment on obesity. We found that there were considerable differences in the effects of the built environment on local obesity rates between older men and women. In other words, older men are more likely to be affected by the built environment than older women. 

The first hypothesis, that there is a greater effect of environmental factors on local obesity for older men than for older women, was supported by the fact that more environmental factors generated higher absolute mean values of local coefficients on obesity for older men than for older women. More specifically, the obesity of older men was affected by the number of sport facilities. This suggests that an increase in the number of sports facilities reduces obesity in older men. Older women were generally more sedentary than older men [48], so they were less likely to recognize easy access to places for physical activity [68]. In addition, the number of fast-food restaurants affected the obesity of older men. Older women were more likely to value healthy eating, as compared to older men, and as a result, were more likely to avoid fast-food restaurants [69].

The second hypothesis, that there is more varying effect of environmental factors on local obesity for older men than for older women, was supported by the wider range of standard deviations of the local significant coefficients on obesity for older men than for older women. More specifically, regional differences were present especially in older men, with respect to fast-food restaurants (in the northeast), sports facilities (in the southeast and north), and intersection density (in the west). Given these findings of more spatial variability of the effects of environmental factors on obesity for older men, we suggest that spatial heterogeneity and gender difference be considered when analyzing obesity. 

With regards to these findings, we suggest several policy implications. It is essential to develop policies that can engage older men with easily accessible exercise programs and provide information for exercise in sport facilities. In addition, policy makers should consider policies which limit the availability of fast-food restaurants, or obligate restaurants to post warnings about the effects of fast-food on weight gain, especially in older men. Given that older women are less affected by the built environment than men, the local government should consider developing social programs for older women. These programs will allow older women to join exercise and activity groups in their neighborhoods with specialized personnel that will help them to develop a healthier routine to promote weight loss, while also helping them create new social contacts. Given these findings of spatial variability of environmental factors and gender difference, we believe policies for obesity prevention should not rest on the assumption of spatial homogeneity and gender indifference, but rather, on gender-specific spatial heterogeneity in local obesity. This approach not only suggests geographically-focused strategies for obesity prevention, but also designating resources and services based on specific demands suitable to spatial characteristics and population groups.

Our study also has limitations, similar to other obesity studies that have been conducted in the past. Local obesity rate was calculated using self-reported weight and height. People tend to report increased heights and lowered weights in the surveys, which can often result in inaccurate BMI calculations. In addition, our study, which covers the entire country, could not include objective measures of the availability of parks at the local level because such data were not available. As the availability of parks is associated with obesity, it seems that future studies will need to take in to account the availability of parks and its effect. Although we included sports facilities as recreational opportunities, future studies will need to differentiate the types of sports facilities, as different types of recreational opportunities can influence obesity differently.

## 5. Conclusions

In this study, we examined the effects of built environment on local obesity of older adults by considering gender differences. We asked two research questions: (1) How does the built environment affect obesity for older adults? (2) Is there gender difference in the effect of the built environment? To examine the research questions, we analyzed data from the 2015 Korea Community Health Survey and used GWR analyses. The empirical analyses showed that environmental factors have stronger effects on local obesity rates for older men than for older women, thereby finding a gender difference in obesity. These results suggest that public health policies should consider the role of the built environment on obesity for older adults, in addition considering gender differences. The findings in this study will provide insights for reducing obesity in older adults, which can contribute to developing efficient public health policies.

## Figures and Tables

**Table 1:** Summary statistics of the explanatory variables.

Variables	N	Mean	S.D.	Min	Max
Local obesity rate for women	217	25.94	5.96	9.19	38.82
Local obesity rate for men	217	21.43	5.13	11.02	38.71
Number of fast-food per 1000 people	217	0.51	0.27	0.06	2.35
Number of sports facilities per 1000 people	217	2.09	6.30	0.71	94.95
Level of land-use mix	217	0.34	0.16	0.07	0.76
Intersection density	217	52.00	47.48	0	298
Employment density	217	1615.48	3744.59	2.72	36,494.80
Percentage of high school graduates	217	24.59	12.63	7.23	73.73
Percentage of basic living recipients	217	3.73	1.78	0.68	9.10

**Table 2:** Summary statistics of local coefficients in geographically weighted regression (GWR) for older women and men.

Variables	Older Women			Older Men		
	OLS		GWR	OLS		GWR
	Coef. (S.E.)	t-stat	Mean	Coef. (S.E.)	t-stat	Mean
Intercept	23.03*** (2.02)	11.39	23.914	17.60*** (1.62)	10.88	17.663
Number of fast-food restaurants per 1000 people	1.58 (1.97)	0.80	0.878	3.71** (1.58)	2.35	3.864
Number of sports facilities per 1000 people	0.05 (0.06)	0.77	0.045	−0.11** (0.05)	−2.32	−0.110
Level of land-use mix	3.72 (2.80)	1.33	0.366	2.71 (2.24)	1.21	2.673
Intersection density	−0.01 (0.01)	−1.47	−0.011	0.01 (0.01)	1.32	0.009
Employment density	−0.00 (0.00)	−0.71	0.000	0.00 (0.00)	−1.28	0.000
Percentage of high school graduates	0.14*** (0.04)	3.54	0.145	0.13*** (0.03)	4.10	0.120
Percentage of basic living recipients	−0.50** (0.24)	−2.09	−0.339	−0.57*** (0.19)	−2.96	–0.574
Akaike Information Criterion (AIC)	1360.60		1344.95	1264.05		1263.75
$\;R^{2}\;$	0.20		0.35	0.31		0.35
Adjusted $R^{2}\;$	0.17		0.26	0.28		0.30
N	217			217		

**Table 3:** Summary statistics of local coefficients in GWR for older men and women.

**Older Women**	**Mean**	**S.D.**	**Min**	**Max**	**Range**
Intercept	23.914	6.17	14.70	31.56	16.86
Number of fast-food restaurant per 1000 people	0.878	4.60	−5.63	9.10	14.73
Number of sports facilities per 1000 people	0.045	0.01	0.02	0.06	0.04
Level of land-use mix	0.366	2.99	−6.21	6.12	12.33
Intersection density	−0.011	0.01	−0.04	0.00	0.04
Employment density	0.000	0.00	−0.00	0.00	0.00
Percentage of high school graduates	0.145	0.08	0.04	0.26	0.24
Percentage of basic living recipients	−0.339	0.22	−0.88	0.14	1.02
**Older Men**	**Mean**	**S.D.**	**Min**	**Max**	**Range**
Intercept	17.663	1.07	16.09	19.18	3.09
Number of fast-food restaurant per 1000 people	3.864	0.58	2.52	4.69	2.17
Number of sports facilities per 1000 people	−0.110	0.01	−0.12	−0.10	0.02
Level of land-use mix	2.673	3.04	−1.72	6.81	8.53
Intersection density	0.009	0.01	0.00	0.01	0.01
Employment density	−0.000	0.00	−0.00	−0.00	0.00
Percentage of high school graduates	0.120	0.01	0.10	0.13	0.03
Percentage of basic living recipients	−0.574	0.04	−0.63	−0.50	0.13

**Table 4:** The number of localities with significant coefficients in GWR for older men and women.

Variables	Older Women	Older Men
Intercept	217	217
Number of fast-food restaurants per 1000 people	61	154
Number of sports facilities per 1000 people	0	217
Level of land-use mix	0	76
Intersection density	15	0
Employment density	4	0
Percentage of high school graduates	132	217
Percentage of basic living recipients	10	217
